# Cleaner anaerobic fermentation and greenhouse gas reduction of crop straw

**DOI:** 10.1128/spectrum.00520-24

**Published:** 2024-06-04

**Authors:** Zhumei Du, Andressa Nakagawa, Jiachen Fang, Roni Ridwan, Wulansih D. Astuti, Ki A. Sarwono, Ahmad Sofyan, Yantyati Widyastuti, Yimin Cai

**Affiliations:** 1 College of Animal Science and Technology, Yangzhou University, Yangzhou, China; 2 Japan International Research Center for Agricultural Sciences (JIRCAS), Tsukuba, Ibaraki, Japan; 3 Faculty of Agriculture and Life Science, Hirosaki University, Hirosaki, Japan; 4 Research Center for Applied Zoology, National Research and Innovation Agency (BRIN), Cibinong, Indonesia; 5 Research Center for Animal Husbandry, National Research and Innovation Agency (BRIN), Cibinong, Indonesia; Institute of Microbiology, Chinese Academy of Sciences, Beijing, China

**Keywords:** cleaner anaerobic fermentation, crop straw resource, greenhouse gas emission source, microbial co-occurrence network, sustainable animal production

## Abstract

Rice anaerobic fermentation is a significant source of greenhouse gas (GHG) emissions, and in order to efficiently utilize crop residue resources to reduce GHG emissions, rice straw anaerobic fermentation was regulated using lactic acid bacteria (LAB) inoculants (FG1 and TH14), grass medium (GM) to culture LAB, and *Acremonim cellulolyticus* (AC). Microbial community, GHG emission, dry matter (DM) loss, and anaerobic fermentation were analyzed using PacBio single-molecule real-time and anaerobic fermentation system. The epiphytic microbial diversity of fresh rice straw was extremely rich and contained certain nutrients and minerals. During ensiling, large amounts of GHG such as carbon dioxide are produced due to plant respiration, enzymatic hydrolysis reactions, and proliferation of aerobic bacteria, resulting in energy and DM loss. Addition of FG1, TH14, and AC alone improved anaerobic fermentation by decreasing pH and ammonia nitrogen content (*P* < 0.05) and increased lactic acid content (*P* < 0.05) when compared to the control, and GM showed the same additive effect as LAB inoculants. Microbial additives formed a co-occurrence microbial network system dominated by LAB, enhanced the biosynthesis of secondary metabolites, diversified the microbial metabolic environment and carbohydrate metabolic pathways, weakened the amino acid metabolic pathways, and made the anaerobic fermentation cleaner. This study is of great significance for the effective utilization of crop straw resources, the promotion of sustainable livestock production, and the reduction of GHG emissions.

**IMPORTANCE:**

To effectively utilize crop by-product resources, we applied microbial additives to silage fermentation of fresh rice straw. Fresh rice straw is extremely rich in microbial diversity, which was significantly reduced after silage fermentation, and its nutrients were well preserved. Silage fermentation was improved by microbial additives, where the combination of cellulase and lactic acid bacteria acted as enzyme-bacteria synergists to promote lactic acid fermentation and inhibit the proliferation of harmful bacteria, such as protein degradation and gas production, thereby reducing GHG emissions and DM losses. The microbial additives accelerated the formation of a symbiotic microbial network system dominated by lactic acid bacteria, which regulated silage fermentation and improved microbial metabolic pathways for carbohydrates and amino acids, as well as biosynthesis of secondary metabolites.

## INTRODUCTION

The increase in the concentration of greenhouse gas (GHG) emitted into the atmosphere by human industrial and agricultural production activities has led to global warming, and natural disasters such as typhoons and hurricanes, as well as secondary disasters such as mudslides and landslides due to rising temperatures, pose a serious threat to the safety of people’s lives and property (1). Agriculture is one of the key GHG-emitting sectors, with livestock industry accounting for 30% of global GHG emissions and ruminants accounting for 5% of global methane (CH_4_) emissions (2). Therefore, how to monitor GHG emissions from agricultural and livestock production and the development of abatement technologies are extremely important for global climate change research (3).

Studies on the behavior of GHG emissions downstream of the livestock processing chain, such as metabolism in the animal’s rumen and intestine, as well as livestock excreta disposal, have been intensively explored (4). However, there is limited information on GHG emissions upstream of this processing chain, such as crop by-product treatment, feed processing, and anaerobic fermentation. In order to ensure a stable supply of livestock feed throughout the year, there is a need to actively utilize local feed resources and to adopt effective feed preparation and storage methods to cope with the global problem of insufficient feed during the dry season in the tropics and during the winter in temperate regions (5). Anaerobic fermentation is considered an ideal method for livestock storage feed preparation, which can effectively utilize agricultural by-product resources to modulate silage, improve palatability and intake of livestock while maintaining no loss of feed components, and promote the productivity of ruminant livestock such as goats and beef cattle. Therefore, anaerobic fermentation technology plays an important role in alleviating feed shortage and promoting sustainable livestock production (6).

Currently, fermented feed and hay are the most important storage feeds for ruminant production in the world (7). Hay is a stored feed made through a drying process, while fermented feed is a high-moisture storage feed prepared through lactic acid fermentation (8). During fermentation, large amounts of carbon dioxide (CO_2_) are produced due to plant respiration, enzymatic hydrolysis reactions, and proliferation by aerobic bacteria and heterofermentative lactic acid bacteria (LAB), resulting in energy and dry matter (DM) losses (9). In particular, plant respiration is the process of converting organic matter (OM) into CO_2_ and water and releasing energy by utilizing the oxygen remaining in the silage material during the early stages of anaerobic fermentation. In anaerobic environments, some species from *Methanobacterium*, *Thiobacillus*, and *Pseudomonas* can produce nitrous oxide (N_2_O) and CH_4_ (10). In addition, *Saccharomyces cerevisiae* breaks down sugar into alcohol and CO_2_; heterofermentative LAB converts sugar into lactic acid and CO_2_ (11); *Clostridia* produces butyric acid and degrades proteins and amino acids into ammonia nitrogen (NH_3_-N) (12). The metabolic pathways of these microorganisms are closely linked to GHG emissions and feed energy losses. During anaerobic fermentation, microorganisms utilize carbohydrates to produce CO_2_, which increases DM losses from feeds. N_2_O and NH_3_-N produced through protein decomposition by microorganisms affect the nutritional value of the feed. The decomposition of carbohydrates and proteins can lead to feed losses of up to 10% (13). In addition, during CH_4_ generation, the complex OM is first decomposed into monosaccharides, amino acids, fatty acids, etc., through the hydrolysis-acidification stage. Then, in the hydrogen and acetic acid production stage, these components are further converted into acetic acid, propionic acid, formic acid, and so on. Finally, in the methanogenesis stage, acetic acid or CO_2_ and hydrogen are converted to CH_4_ by methanogenic bacteria. These chemical reactions not only reduce the fermentation quality, nutritional value, and energy of feeds but also increase the cost of feed production and affect the profitability of farmers (14).

Rice is one of the most important food crops globally, with rice-growing soils covering 115 million hectares of the earth’s surface and consumed by more than 50% of the world’s population (15). During the rice harvesting season, a large amount of rice straw is produced every year. Some of these rice straws are widely used as feed for ruminants, household fuel, substrate for edible mushrooms, handicrafts, compost and biogas fermentation materials, among others (16). However, in some rice-producing countries, post-harvest waste rice straw is treated by incineration or ploughed back to the field (17). Global warming triggered by GHG, such as CO_2_, produced by burning rice straw, can lead to frequent natural disasters, which can affect human ecology environment and the safety of life and property (18). Therefore, effective utilization of rice straw resources for making high-quality fermented feed is important for curbing GHG emissions and global warming, as well as for promoting sustainable development of the livestock sector. Usually, microbial additives may promote anaerobic fermentation and help reduce DM and nutrient losses from field to feed (11). The LAB and cellulolytic enzyme additives were selected for this study, which have enzyme-bacteria synergistic effects during anaerobic fermentation, with cellulolytic enzyme-decomposing carbohydrates in the material and providing a fermentation substrate for LAB to promote anaerobic fermentation. The LAB inhibits harmful microorganisms in anaerobic environments, which may include GHG-producing bacteria. In this study, GHG emissions, DM loss, and fermentation characteristics during anaerobic fermentation of rice straw were studied using microbial additives.

## RESULTS

The microbial population, pH, lactic acid buffering capacity (LBC), chemical composition, protein conposition, energy, and macro-mineral of rice straw material are shown in Table 1. The pH and LBC contents of rice straw were below 6 and above 600 meq/kg of DM, respectively. The DM and OM were higher than 30% and 80% of DM, respectively. The crude protein (CP) and ether extract (EE) contents of rice straw were lower than 4% and 2.5% of DM, respectively. The neutral detergent fiber (NDF), acid detergent fiber (ADF), and acid detergent lignin (ADL) contents of rice straw exceeded 63%, 37%, and 1% of DM, respectively. The protein composition including non-protein nitrogen (NPN), true protein (TP), and neutral detergent insoluble protein (NDIP) were 1%–3%, and the energy including net energy for maintenance (NEm), net energy for lactation (NEl), and net energy for gain (NEg) were below 1 MJ/kg of DM. The calcium (Ca), phosphorous (P), and magnesium (Mg) contents were less than 0.4 mg/kg, and the potassium (K) content was higher than 0.9 mg/kg based on DM. For the microbial population, the LAB, yeast, and mold counts ranged from 10^2^ to 10^3^ colony-forming unit (CFU)/g of fresh matter (FM), the aerobic bacteria and coliform bacteria counts were 10^6^–10^7^ CFU/g of FM.

**TABLE 1:** Microbial population and chemical composition of rice straw before ensiling^*a*^

Item	Rice straw
Microbial population (CFU/g of FM)	
Lactic acid bacteria	1.3 × 10^4^
Aerobic bacteria	6.3 × 10^6^
Coliform bacteria	4.7 × 10^7^
Yeast	1.2 × 10^2^
Mold	2.4 × 10^2^
pH	5.73 ± 0.13
LBC (meq/kg of DM)	606.83 ± 25.57
Chemical composition	
DM (%)	31.50 ± 1.40
OM (% DM)	81.79 ± 0.97
CP (% DM)	3.89 ± 0.05
EE (% DM)	2.42 ± 0.17
NDF (% DM)	63.01 ± 0.75
ADF (% DM)	37.55 ± 0.43
ADL (% DM)	1.45 ± 0.20
Protein composition (% DM)	
NPN	1.29 ± 0.03
TP	2.60 ± 0.18
NDIP	1.67 ± 0.08
Energy (MJ/kg DM)	
NEm	0.99 ± 0.08
NEl	0.95 ± 0.10
NEg	0.44 ± 0.07
Macro-mineral (mg/kg DM)	
Calcium	0.39 ± 0.07
Phosphorous	0.11 ± 0.33
Magnesium	0.19 ± 0.04
Potassium	0.99 ± 0.10

^*a*^ Data are mean ± standard deviation of three samples. ADF, acid detergent fiber; ADL, acid detergent lignin; CFU, colony-forming unit; CP, crude protein; DM, dry matter; EE, ether extract; FM, fresh matter; LBC, lactic acid buffering capacity; NDF, neutral detergent fiber; NDIP, neutral detergent insoluble protein; NEg, net energy for gain; NEl, net energy for lactation; NEm, net energy for maintenance; NPN, non-protein nitrogen; OM, organic matter; TP, true protein.

Experiments A and B were designed to investigate the mutual synergistic effects of cellulolytic enzyme with *Lactiplantibacillus plantarum* and *Lacticaseibacillus casei*, respectively. The chemical composition, protein composition, energy, and macro-mineral of rice straw anaerobic fermentation after 60 days of ensiling are shown in Table 2. The DMs of anaerobic fermentation in experiment A and B treatments ranged from 27% to 31%. There were no significant differences in OM, CP, EE, ADL, NPN, TP, NDIP, NEm, NEl, NEg, Ca, P, Mg, and K contents of anaerobic fermentation in the two experiments. In experiment A, when anaerobic fermentation was treated with FG1, *Acremonim cellulolyticus* (AC), FG1 + AC, and FM + AC, the NDF content was significantly (*P* < 0.001) reduced compared to those of the control, and the FM + AC treatment had the lowest value. In experiment B, when anaerobic fermentation was treated with TM, AC, TH14 + AC, TM + AC, the NDF content was significantly (*P* < 0.001) reduced compared to those of the control, and the TM + AC treatment had the lowest value. The ADF content in the FM + AC treatment in experiment A, and AC, TH14 + AC and TM + AC treatments in experiment B were significantly (*P* = 0.004 and <0.001) reduced compared to that of each control.

**TABLE 2:** Chemical composition of rice straw fermented feed after 60 days of ensiling^*a*^^,^^*b*^

Item	Experiment A								Experiment B							
	Control	FG1	FM	AC	FG1 + AC	FM + AC	SEM	*P* value	Control	TH14	TM	AC	TH14 + AC	TM + AC	SEM	*P* value
Chemical composition																
DM (%)	28.40 ± 1.06ab	28.93 ± 0.50a	27.47 ± 0.91b	28.13 ± 0.40ab	27.63 ± 0.45ab	27.80 ± 0.56ab	0.40	0.17	28.03 ± 0.70	30.10 ± 0.53	28.30 ± 0.17	30.17 ± 1.70	29.23 ± 1.33	29.27 ± 1.79	0.69	0.22
OM (% DM)	79.78 ± 0.32	80.33 ± 0.12	80.06 ± 0.43	79.98 ± 0.27	79.97 ± 0.46	80.35 ± 0.10	0.18	0.25	79.92 ± 0.27b	80.77 ± 0.44a	79.94 ± 0.55b	79.63 ± 0.09b	80.25 ± 0.40ab	79.91 ± 0.21b	0.21	0.03
CP (% DM)	4.13 ± 0.03	4.15 ± 0.14	3.97 ± 0.04	4.07 ± 0.12	4.07 ± 0.14	4.02 ± 0.02	0.05	0.26	4.01 ± 0.11ab	4.09 ± 0.08ab	3.85 ± 0.19b	4.18 ± 0.16a	4.20 ± 0.10a	4.04 ± 0.10ab	0.07	0.05
EE (% DM)	1.31 ± 0.08	2.79 ± 0.21	2.01 ± 0.09	2.14 ± 0.20	2.29 ± 0.15	2.66 ± 0.10	0.70	0.71	2.51 ± 0.13	2.92 ± 0.09	2.95 ± 0.08	3.66 ± 0.17	2.97 ± 0.15	2.39 ± 0.18	0.69	0.83
NDF (% DM)	64.83 ± 0.26a	63.02 ± 0.42b	65.59 ± 0.16a	60.92 ± 1.03c	60.10 ± 1.41c	59.40 ± 1.06c	0.50	<0.001	62.06 ± 0.61a	62.38 ± 0.42a	60.88 ± 0.48b	59.68 ± 0.23b	59.20 ± 0.67b	58.57 ± 0.27c	1.43	0.04
ADF (% DM)	38.15 ± 0.73ab	38.16 ± 0.57ab	39.07 ± 0.05a	36.80 ± 0.38bc	36.92 ± 0.26bc	36.21 ± 0.67c	0.43	<0.001	38.75 ± 0.49a	37.94 ± 0.62a	37.60 ± 0.43a	34.89 ± 0.22b	34.36 ± 0.20b	33.43 ± 0.51b	0.72	<0.001
ADL (% DM)	1.99 ± 0.04	2.06 ± 0.05	1.48 ± 0.08	1.44 ± 0.08	1.33 ± 0.13	1.49 ± 0.06	0.47	0.82	1.30 ± 0.09	2.11 ± 0.07	2.14 ± 0.11	1.99 ± 0.08	1.59 ± 0.16	1.30 ± 0.80	0.50	0.69
Protein composition (% DM)																
NPN	1.08 ± 0.09	0.89 ± 0.09	0.89 ± 0.08	0.91 ± 0.05	0.74 ± 0.03	0.87 ± 0.09	0.11	0.45	0.93 ± 0.09	0.99 ± 0.05	0.91 ± 0.07	0.89 ± 0.08	0.90 ± 0.08	0.84 ± 0.04	0.12	0.35
TP	3.05 ± 0.09b	3.45 ± 0.05a	3.08 ± 0.06b	3.16 ± 0.04ab	3.32 ± 0.07ab	3.15 ± 0.04ab	0.10	0.10	3.08 ± 0.11	3.10 ± 0.11	2.94 ± 0.06	3.28 ± 0.09	3.30 ± 0.08	3.20 ± 0.04	0.26	0.21
NDIP	1.26 ± 0.05a	1.34 ± 0.06a	1.17 ± 0.06ab	1.16 ± 0.04ab	1.06 ± 0.10b	1.28 ± 0.09a	0.06	0.04	1.22 ± 0.15	1.25 ± 0.12	1.17 ± 0.08	1.21 ± 0.18	1.23 ± 0.06	1.11 ± 0.07	0.06	0.73
Energy (MJ/kg DM)																
NEm	0.89 ± 0.04b	0.98 ± 0.12ab	0.95 ± 0.06ab	0.98 ± 0.02ab	1.02 ± 0.02a	1.05 ± 0.09a	0.03	0.12	1.01 ± 0.08	0.99 ± 0.08	0.97 ± 0.06	1.02 ± 0.07	1.04 ± 0.09	1.06 ± 0.06	0.04	0.68
NEl	0.87 ± 0.03b	0.94 ± 0.10ab	0.92 ± 0.05ab	0.95 ± 0.02ab	0.98 ± 0.02a	1.01 ± 0.08a	0.03	0.13	0.97 ± 0.06	0.95 ± 0.07	0.94 ± 0.05	0.98 ± 0.06	1.00 ± 0.06	1.01 ± 0.05	0.03	0.66
NEg	0.35 ± 0.04b	0.43 ± 0.11ab	0.40 ± 0.05ab	0.43 ± 0.02ab	0.47 ± 0.02a	0.50 ± 0.09a	0.03	0.13	0.46 ± 0.07	0.44 ± 0.08	0.42 ± 0.06	0.47 ± 0.07	0.49 ± 0.08	0.51 ± 0.06	0.03	0.31
Macro-mineral (g/kg DM)																
Calcium	0.36 ± 0.08	0.36 ± 0.09	0.31 ± 0.07	0.34 ± 0.09	0.33 ± 0.08	0.34 ± 0.10	0.05	0.98	0.36 ± 0.10	0.38 ± 0.08	0.29 ± 0.02	0.32 ± 0.04	0.29 ± 0.05	0.28 ± 0.06	0.01	0.08
Phosphorous	0.12 ± 0.01a	0.11 ± 0.01b	0.12 ± 0.01ab	0.12 ± 0.01a	0.12 ± 0.01ab	0.12 ± 0.01a	0.01	0.02	0.12 ± 0.01a	0.10 ± 0.01b	0.12 ± 0.01ab	0.11 ± 0.01ab	0.12 ± 0.01ab	0.12 ± 0.01ab	0.01	0.37
Magnesium	0.16 ± 0.01	0.16 ± 0.02	0.16 ± 0.02	0.17 ± 0.03	0.16 ± 0.02	0.17 ± 0.02	0.01	0.93	0.17 ± 0.02	0.17 ± 0.02	0.14 ± 0.02	0.14 ± 0.12	0.16 ± 0.03	0.16 ± 0.02	0.07	0.27
Potassium	2.18 ± 0.15	2.15 ± 0.19	2.21 ± 0.06	2.15 ± 0.10	2.14 ± 0.08	2.11 ± 0.08	0.07	0.93	2.17 ± 0.07	1.96 ± 0.08	2.11 ± 0.10	2.14 ± 0.15	2.07 ± 0.16	1.98 ± 0.14	0.03	0.42

^*a*^ Data are mean ± standard deviation of three samples. a–c denotes statistical difference for each experiment in the same row (*P* < 0.05).

^*b*^ FG1, inoculant *Lactiplantibacillus plantarum*; FM, strain FG1 cultured in grass medium; AC, *Acremonium cellulase*; TH14, inoculant *Lacticaseibacillus casei*; TM, strain TH14 cultured in grass medium; DM, dry matter; OM, organic matter; CP, crude protein; EE, ether extract; NDF, neutral detergent fiber; ADF, acid detergent fiber; ADL, acid detergent lignin; NPN, non-protein nitrogen; TP, true protein; NDIP, neutral detergent insoluble protein; NEm, net energy for maintenance; NEl, net energy for lactation; NEg, net energy for gain.

The microbial population and fermentation quality of rice straw anaerobic fermentation in experiments A and B are shown in Table 3. After 60 days of anaerobic fermentation, LAB was the dominant population of rice straw anaerobic fermentation in both experiments, with 10^6^–10^8^ CFU/g of FM. The aerobic bacteria and coliform bacteria in both control anaerobic fermentation reached 10^5^ and 10^4^ CFU/g of FM, while these bacteria in other anaerobic fermentations were less than 10^4^ and below the detectable levels (<10^2^ CFU/g of FM), respectively. The yeast counts ranged from 10^2^ to 10^5^ CFU/g of FM, and the molds were below the detectable levels (<10^2^ CFU/g of FM) in all anaerobic fermentation. Compared to the control anaerobic fermentation, the additive-treated anaerobic fermentations in experiments A and B were well preserved, with lower (*P* < 0.05) pH and contents of acetic acid and NH_3_-N, and higher (*P* < 0.05) lactic acid content. The grass medium (GM) + AC-treated anaerobic fermentation in both experiments had the best fermentation quality with higher (*P* < 0.001) lactic acid and lower (*P* < 0.001) pH and NH_3_-N content than other anaerobic fermentations. The propionic acid and butyric acid contents were below the detectable level (<0.001% of FM).

**TABLE 3:** Microbial population and fermentation quality of rice straw fermented feed^*a*^^,^^*b*^

Item	Experiment A								Experiment B							
	Control	FG1	FM	AC	FG1 + AC	FM + AC	SEM	*P* value	Control	TH14	TM	AC	TH14 + AC	TM + AC	SEM	*P* value
Microbial population (CFU/g of FM)																
LAB	8.23 ± 0.20ab	8.52 ± 0.15a	8.11 ± 0.13bc	7.83 ± 0.16c	6.73 ± 0.24d	6.11 ± 0.07e	0.10	<0.001	7.70 ± 0.13a	7.36 ± 0.10b	7.76 ± 0.10a	7.34 ± 0.07b	6.81 ± 0.07c	6.51 ± 0.08d	0.05	<0.001
Aerobic bacteria	5.74 ± 0.13a	4.68 ± 0.14b	4.34 ± 0.13c	4.61 ± 0.09b	2.48 ± 0.14e	2.86 ± 0.13d	0.07	<0.001	5.79 ± 0.23a	4.90 ± 0.11b	4.00 ± 0.10d	4.58 ± 0.08c	2.72 ± 0.15f	3.18 ± 0.14e	0.08	<0.001
Coliform bacteria	4.38 ± 0.21	ND	ND	ND	ND	ND	ND	ND	4.59 ± 0.14	ND	ND	ND	ND	ND	ND	ND
Yeast	4.76 ± 0.16a	3.43 ± 0.20c	3.28 ± 0.09c	4.72 ± 0.15a	4.04 ± 0.07b	3.45 ± 0.20c	0.09	<0.001	4.81 ± 0.16ab	4.18 ± 0.11c	3.83 ± 0.17d	4.86 ± 0.16a	4.56 ± 0.15b	4.23 ± 0.17c	0.09	<0.001
Mold	ND	ND	ND	ND	ND	ND	ND	ND	ND	ND	ND	ND	ND	ND	ND	ND
Fermentation quality																
pH	5.01 ± 0.05a	4.63 ± 0.04b	4.59 ± 0.09b	4.56 ± 0.09b	4.40 ± 0.07bc	4.19 ± 0.03c	0.07	<0.001	4.97 ± 0.07a	4.57 ± 0.14b	4.46 ± 0.04b	4.47 ± 0.02b	4.13 ± 0.02c	4.04 ± 0.05c	0.04	<0.001
Lactic acid (% FM)	0.52 ± 0.05d	0.74 ± 0.10cd	0.85 ± 0.06bc	0.89 ± 0.04abc	1.03 ± 0.09ab	1.14 ± 0.05a	0.08	<0.001	0.66 ± 0.07c	0.88 ± 0.04b	0.95 ± 0.04ab	0.98 ± 0.03ab	1.11 ± 0.08ab	1.17 ± 0.03a	0.07	<0.001
Acetic acid (% FM)	0.47 ± 0.05	0.36 ± 0.04	0.45 ± 0.04	0.38 ± 0.03	0.37 ± 0.11	0.41 ± 0.06	0.03	0.15	0.38 ± 0.06	0.46 ± 0.06	0.49 ± 0.06	0.40 ± 0.04	0.50 ± 0.04	0.52 ± 0.05	0.05	0.50
Propionic acid (% FM)	ND	ND	ND	ND	ND	ND	ND	ND	ND	ND	ND	ND	ND	ND	ND	ND
Butyric acid (% FM)	ND	ND	ND	ND	ND	ND	ND	ND	ND	ND	ND	ND	ND	ND	ND	ND
NH_3_-N (g/kg DM)	0.61 ± 0.03a	0.44 ± 0.04c	0.52 ± 0.05b	0.36 ± 0.03d	0.33 ± 0.03d	0.23 ± 0.03e	0.02	<0.001	0.52 ± 0.03	0.41 ± 0.01	0.36 ± 0.04	0.35 ± 0.03	0.29 ± 0.03	0.25 ± 0.02	0.01	<0.001

^*a*^ Data are mean ± standard deviation of three samples. a–f denote statistical difference for each experiment in the same row (*P* < 0.05).

^*b*^ FG1, inoculant *Lactiplantibacillus plantarum*; FM, strain FG1 cultured in grass medium; AC, *Acremonium cellulase*; TH14, inoculant *Lacticaseibacillus casei*; TM, strain TH14 cultured in grass medium; CFU, colony-forming unit; FM, fresh matter; LAB, lactic acid bacteria; ND, not detected; DM, dry matter; NH_3_-N, ammonia nitrogen.

**Fig 1:**
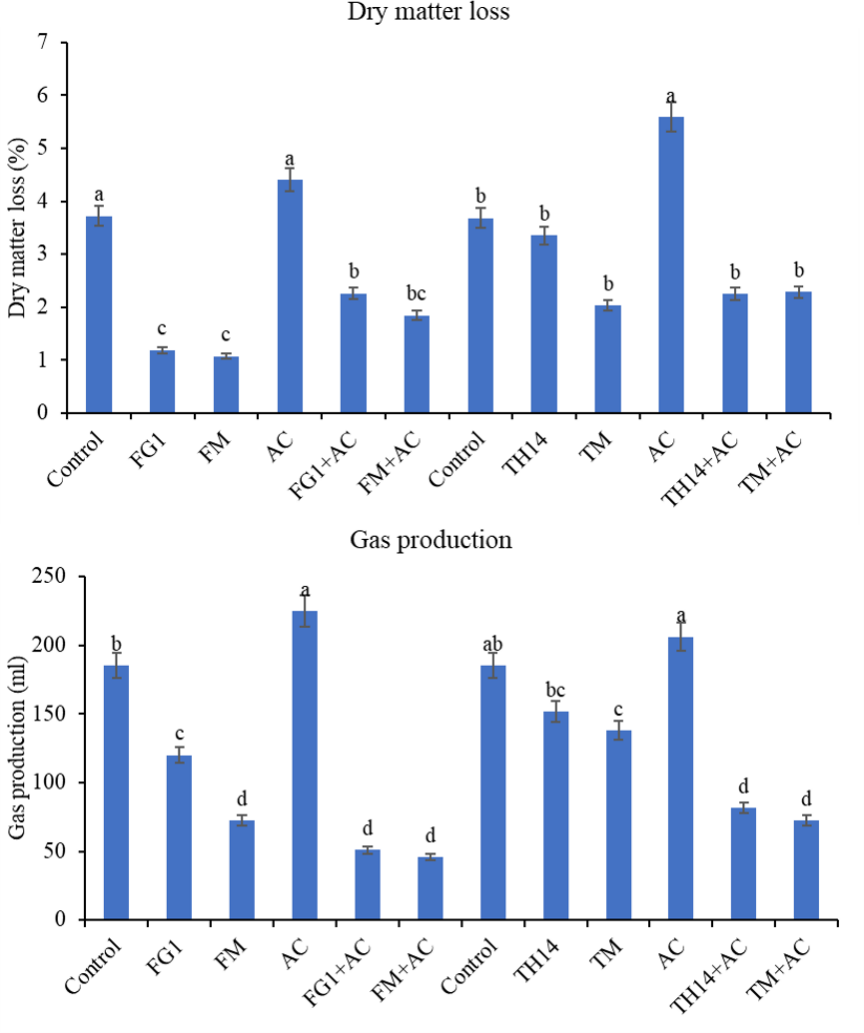
Dry matter loss and gas emission of rice straw fermented feed. AC, *Acremonium cellulase*; FG1, inoculant *Lactiplantibacillus plantarum*; FM, strain FG1 cultured in grass medium; TH14, inoculant *Lacticaseibacillus casei*; TM, strain TH14 cultured in grass medium.

The gas production and DM loss of rice straw anaerobic fermentation after 60 days of ensiling in experiments A and B are shown in Fig. 1. Compared to the control anaerobic fermentation, the gas production and DM loss increased (*P* < 0.05) in the AC treatment but decreased (*P* < 0.05) in the other treatments. The FM in experiment A and the TM in experiment B had the lowest level of DM loss, and the FM + AC in experiment A and TM + AC in experiment B had the lowest level of gas production.

**Fig 2:**
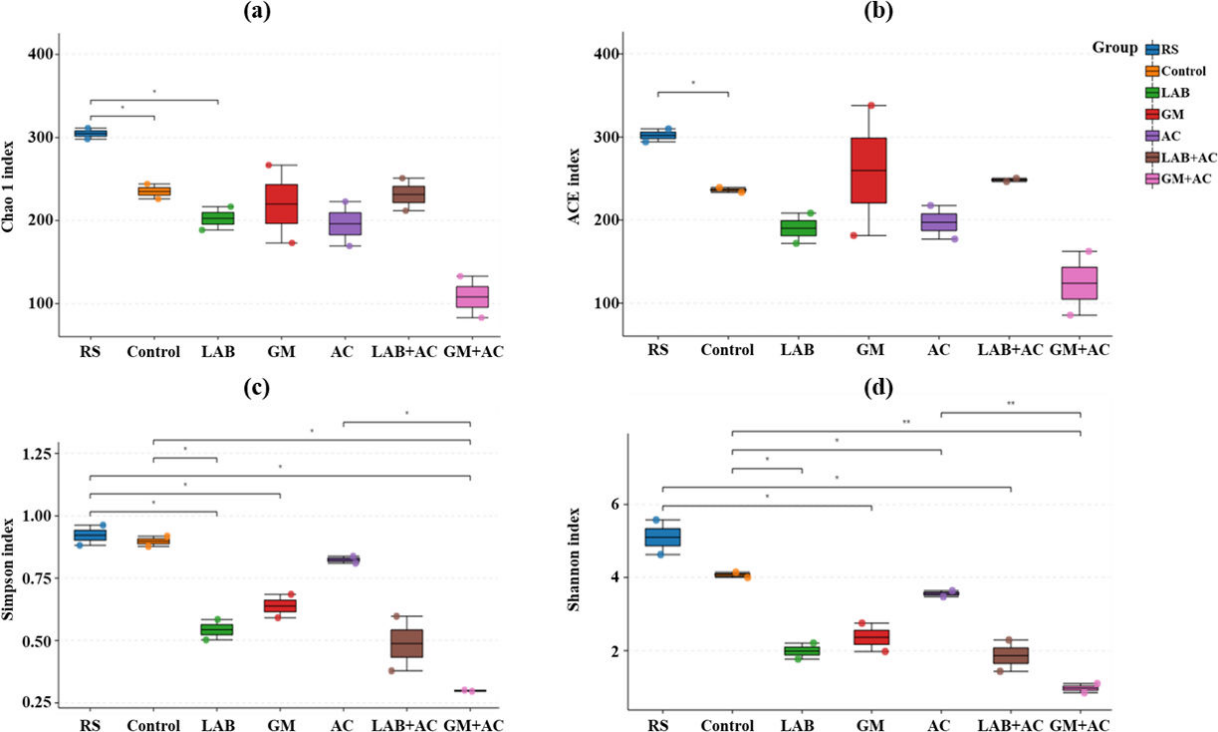
General information of alpha diversity analysis of rice straw material and fermented feed (a, Chao 1 index; b, ACE index; c, Simpson index; d, Shannon index). AC, *Acremonium cellulase*; ACE, abundance-based coverage estimator; GM, grass medium including FM and TM; LAB, lactic acid bacteria including FG1 and TH14; RS, rice straw. Data are averages of the corresponding treatments in experiments A and B. *P < 0.05, **P < 0.01.

General information of alpha diversity indexes of rice straw before and after ensiling is shown in Fig. 2. The corresponding treatments of experiments A and B showed similar alpha diversity indexes, and the average values were taken to draw the alpha diversity plots. Compared with the material, the control and LAB treatments reduced (*P* < 0.05) the Chao 1 index (Fig. 2a). The control anaerobic fermentation reduced (*P* < 0.05) the abundance-based coverage estimator (ACE) index compared with the material (Fig. 2b). The Chao 1 and ACE indexes for the other treatments did not differ significantly. Simpson and Shannon indexes were reduced (*P* < 0.05) for all additive treatments compared to control anaerobic fermentation, with the GM + AC treatment having the lowest indexes (Fig. 2c and d).

**Fig 3:**
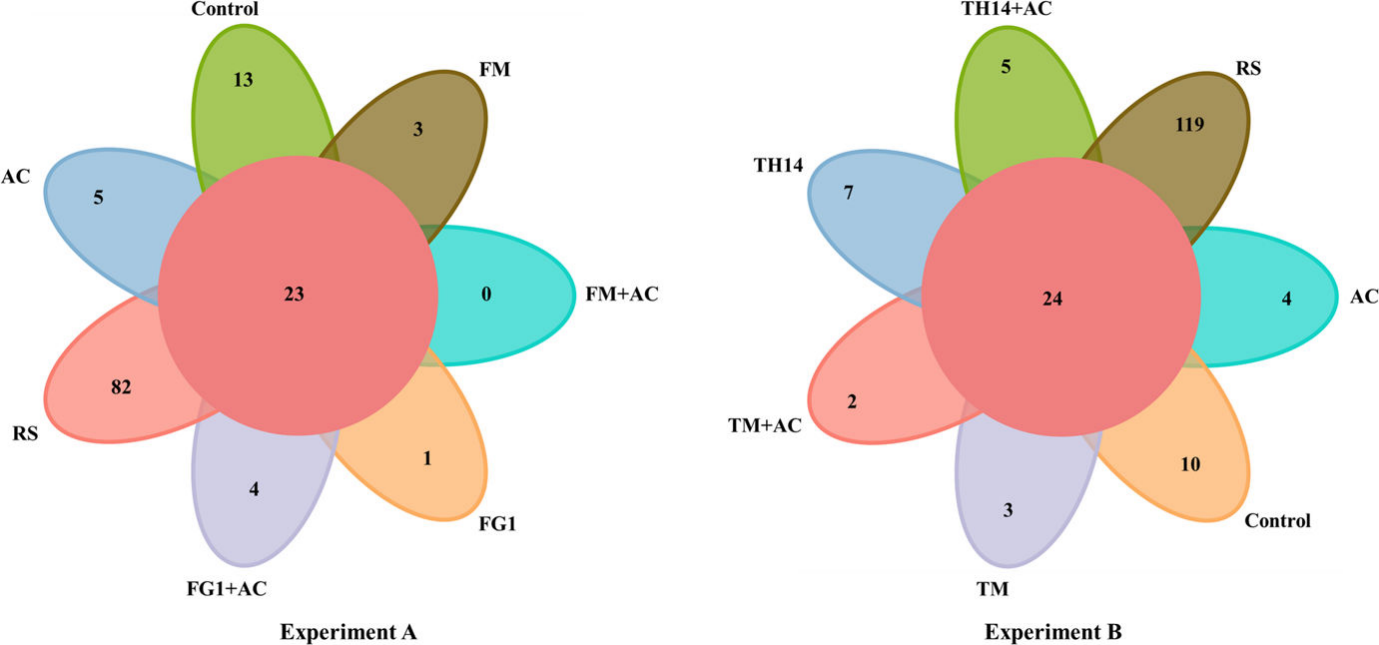
Venn diagram of OTU number in experiment A and experiment B of rice straw before and after ensiling. AC, *Acremonium cellulase*; FG1, inoculant *Lactiplantibacillus plantarum*; FM, strain FG1 cultured in grass medium; OTU, operational taxonomic unit, with 97% of 16S RNA sequence identity; TH14, inoculant *Lacticaseibacillus casei*; TM, strain TH14 cultured in grass medium.

A Venn diagram of the operational taxonomic unit (OTU) numbers in experiments A and B of rice straw materials and anaerobic fermentation is shown in Fig. 3. The dominant microbiome of straw material and anaerobic fermentation contained 23 and 24 shared OTUs, and 0–82 and 2–119 unique OTUs, respectively, in experiments A and B. In both experiments, unique OTUs of bacteria identified by 16S RNA sequence were reduced in all anaerobic fermentation compared to rice straw material, and the number of unique OTU was lower than that of the control anaerobic fermentation for all additive-treated anaerobic fermentation. Both GM + AC-treated anaerobic fermentations showed the respective lowest number of unique OTU in each experiment.

**Fig 4:**
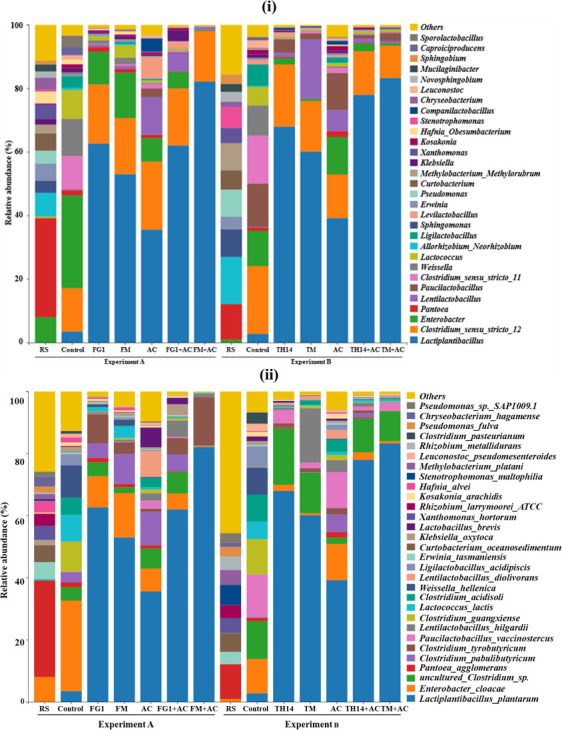
Relative abundance of the bacterial community* associated with material and fermented feed of rice straw. *Bacteria were identified as top 30 genus (i) and species (ii) levels. AC, *Acremonium cellulase*; FG1, inoculant *Lactiplantibacillus plantarum*; FM, strain FG1 cultured in grass medium; RS, RS, rice straw; TH14, inoculant *Lacticaseibacillus casei*; TM, strain TH14 cultured in grass medium.

The relative abundances of the bacterial communities at the (i) genus and (ii) species levels of rice straw materials and anaerobic fermentation in the experiments A and B are shown in Fig. 4. Before ensiling, the dominant genus were *Enterobacter* and *Pantoea*, and their species were *Enterobacter cloacae* and *Pantoea agglomerans*. After ensiling, *E. cloacae* was the dominant species, there are large numbers of *L. plantarum* and uncultured *Clostridium* sp. in the both control anaerobic fermentation. The *L. plantarum* spp. were the dominant LAB in all additive-treated anaerobic fermentations with higher relative abundance than that of control anaerobic fermentation, and the highest relative abundances were presented in each GM + AC-treated anaerobic fermentation. In experiment A, the relative abundance of *L. plantarum* was in the order of FM + AC, FG1, FG1 + AC, FM, and AC. In experiment B, the relative abundance was in the order of TM + AC, TH14 + AC, TH14, TM, and AC.

**Fig 5:**
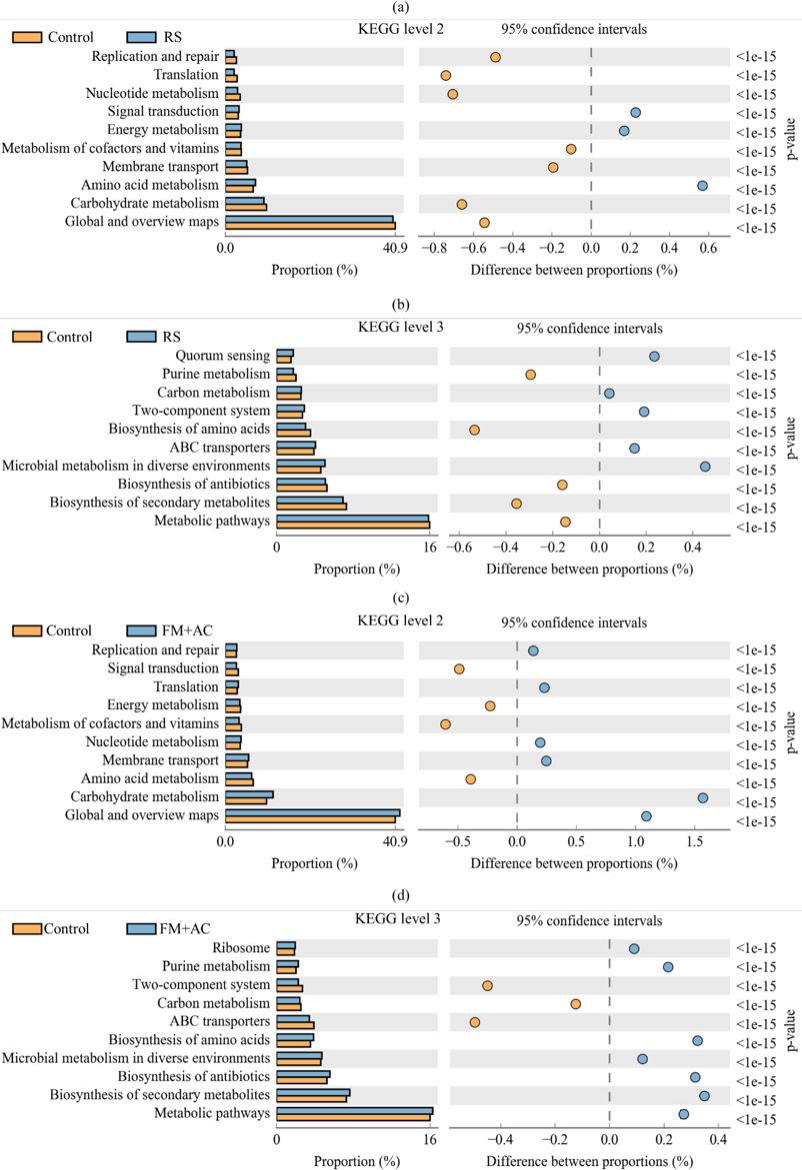
Impacted KEGG metabolism pathway of anaerobic fermentation on the second and third levels. (**a**) RS material and control fermented feed at the level 2; (**b**) RS material and control fermented feed at the level 3; (**c**) control and FM + AC-treated fermented feed at the level 2; (**d**) control and FM + AC-treated fermented feed at the level 3. AC, *Acremonium cellulase*; FG1, inoculant *Lactiplantibacillus plantarum*; FM, strain FG1 cultured in grass medium; RS, rice straw.

Kyoto Encyclopedia of Genes and Genomes (KEGG) is a database resource for large-scale molecular data sets generated by genome sequencing and other high-throughput experimental techniques to understand the high-level function and utility of biological ecosystems. Modulation of the KEGG metabolic pathway by microbial addition can directly affect metabolites and fermentation quality in anaerobic fermentation. The impacted KEGG metabolism pathway of anaerobic fermentation and microbial additive on the second and third levels is shown in Fig. 5. In Fig. 5a, the main proportion of KEGG secondary profiles in rice straw material and control anaerobic fermentation was global and overview maps (GOM), carbohydrate metabolism, and amino acid metabolism. The proportion of GOM metabolism and carbohydrate metabolism pathway was higher (*P* < 0.05), and the proportion of amino acid metabolism pathways was lower (*P* < 0.05) in control anaerobic fermentation than in material. In Fig. 5b, metabolic pathways, biosynthesis of secondary metabolites and microbial metabolism in diverse environments were the main metabolic categories at the KEGG level three for both rice straw material and control anaerobic fermentation. Metabolic pathways and biosynthesis of secondary metabolites were higher (*P* < 0.05), and microbial metabolism in diverse environments was lower (*P* < 0.05) in control anaerobic fermentation compared to rice straw material. In Fig. 5c, GOM, carbohydrate metabolism, and amino acid metabolism were the predominant metabolic categories of KEGG level 2 in control and FM + AC-treated anaerobic fermentation. The proportion of GOM metabolism and carbohydrate metabolism pathways was higher (*P* < 0.05), and the amino acid metabolism pathway was lower (*P* < 0.05) in FM + AC-treated anaerobic fermentation compared to the control. In Fig. 5d, metabolic pathways, biosynthesis of secondary metabolites, and microbial metabolism in a diverse environment were the main metabolic categories of KEGG level 3 in the control and FM + AC-treated anaerobic fermentation. Metabolic pathways, biosynthesis of secondary metabolites, and microbial metabolism in a diverse environment were all higher in KEGG level 3 in FM + AC-treated anaerobic fermentation than in the control.

**Fig 6:**
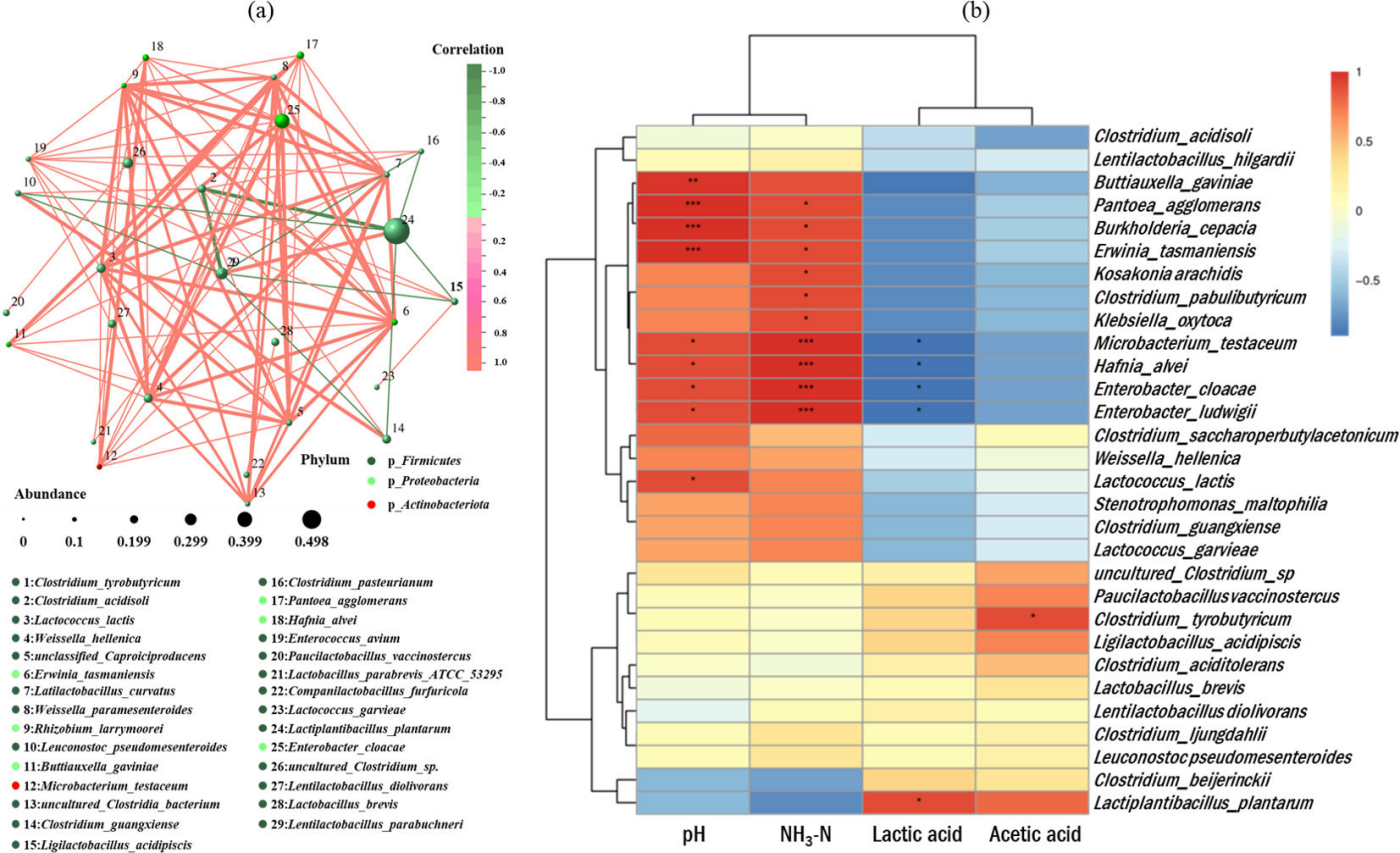
Correlation network (**a**) and correlation analysis (**b**) between bacterial community and terminal fermentation product at species level. NH_3_-N, ammonia nitrogen.

The Fig. 6a shows the correlation analyses of microbial network related to anaerobic fermentation at the species level. The most abundant species in rice straw anaerobic fermentation was *L. plantarum*, which was positively correlated with *Latilactobacillus curvatus* of the Firmicutes phylum and negatively correlated with *Clostridium acidophilum* of the Firmicutes phylum.

Correlation heat map and hierarchical cluster analysis between bacterial community and terminal fermentation products in the rice straw anaerobic fermentation are shown in Fig. 6b. The pH and NH_3_-N were positively correlated with *P. agglomerans*, *Burkholderia cepacia*, and *E. cloacae*. Also, NH_3_-N was positively correlated with *Clostridium pabulibutyricum*. Lactic acid was strongly and positively correlated with *L. plantarum* and negatively correlated with *E. cloacae*. Acetic acid was positively correlated with *Clostridium tyrobutyricum*.

## DISCUSSION

### Chemical composition of rice straw

Rice is grown in more than 100 countries in the world, and 90% of the total global rice production comes from Asia (19). Feed shortage is an important factor limiting the development of animal husbandry in the world (20). The main sources of feed for ruminants are forage crop, grass, and crop by-product (21). Rice straw is preserved as hay by drying it naturally in the field and is used for feeding cattle, sheep, and goats during the dry season, when there is a shortage of feed (5). As shown in Table 1, fresh rice straw contains a certain amount of nutritional values such as CP, EE, energy, and minerals in addition to rich fiber content, indicating that rice straw is one of the important feed resources for ruminants.

Fresh rice straw can be prepared as stored feeds by anaerobic fermentation. The moisture, LBC, and LAB content of rice straw affect the quality of fermented feed (22). The optimum moisture condition for fermented feed is usually 60%–70%. Higher moisture of material tends to cause butyric acid fermentation, which reduces the quality of fermented feed (23). On the other hand, low moisture of material is not conducive to the formation of high-density conditions and the removal of residual air, which can lead to the growth of molds and other aerobic bacteria, thus causing deterioration of fermented feed (24). The moisture of the rice straw used in this experiment just ranged from 60% to 70%, which is within the suitable moisture range for anaerobic fermentation. Therefore, no means of moisture adjustment such as wilting of the material is required in this study, and fresh material can be used directly for preparation (25). The LBC of forage is affected by cations such as phosphorus and magnesium in the material, and if the LBC of forage is higher than 1,000 meq/kg of DM, it will hinder the pH reduction during ensiling (23). The LBC of rice straw in this study was lower than 610 meq/kg of DM, which has a similar LBC level to that of corn stover and is significantly lower than that of leguminous grasses such as alfalfa (7); thus, the rice straw material used in this experiment is suitable for anaerobic fermentation. In addition, there is a diversity of epiphytic microorganisms in the material that constrain each other and influence anaerobic fermentation. In order to prepare high-quality fermented feed, at least 10^5^ CFU/g FM of LAB is required in the material (26). The number, fermentation type, and lactic acid production capacity of LAB in the material will affect the anaerobic fermentation. In this experiment, the LAB counts detected in rice straw was lower than 10^3^ CFU/g of FM, while the counts of aerobic bacteria and coliform bacteria, which are not favorable to anaerobic fermentation, were higher than 10^6^ CFU/g of FM and formed a dominant microbial community. This suggests that it is necessary to use microbial additives to regulate the microbial population related to anaerobic fermentation of rice straw.

### Fermentation characteristics of rice straw anaerobic fermentation

In this study, although the moisture and LBC in the rice straw was suitable for anaerobic fermentation, the low LAB counts and the fact that coliform bacteria and aerobic bacteria were the dominant microbial communities resulted in poor fermentation, as evidenced by low lactic acid content, high pH, and high numbers of harmful bacteria in the control. This may be due to the low epiphytic LAB count and fermentation capacity, which could not produce enough lactic acid to reduce pH and inhibit the growth of undesirable microorganisms during ensiling (27). The addition of either LAB or cellulase alone improved the fermentation quality of rice straw; their combination especially showed a synergistic bacterial-enzymatic effect. The two LAB species used in this study were *L. plantarum* and *L. casei*, which belong to the homofermentative type and can grow vigorously in anaerobic environment and acidic conditions with low pH (28). This suggests that these LAB can produce large amounts of lactic acid during fermentation of rice straw and improve the quality of fermentation. In addition, the cellulase used in this study was produced by cellulolytic mold (*Acremonium cellulolyticus*), including endoglucanase, fiber biohydrolase, and β-glucosidase (6). This enzyme breaks down the plant cell wall and releases the cell contents, thereby reducing the cellulose content of the rice straw (Table 2). The enzyme degrades water-soluble cellulose into water-soluble carbohydrate (WSC) and promotes anaerobic fermentation. Cellulase can provide sufficient fermentation substrate for LAB, which use these sugars to produce lactic acid. Therefore, the combination of these two additives can effectively bring out the synergistic effect of bacteria-enzymes during ensiling (Table 3).

### The GHG emissions and DM loss from rice straw fermented feed

Livestock is one of the main sectors of GHG emissions (29). Currently, the world’s research on GHG in animal husbandry mainly focuses on animal intestinal tubes and livestock composting processes (30, 31), while the GHG emissions during anaerobic fermentation have not yet been thoroughly and systematically studied. Therefore, the development of GHG monitoring and emission reduction technologies for fermented feed is an important research topic in the field of animal husbandry.

Feed fermentation can produce large amounts of GHG during two biochemical reactions of aerobic and anaerobic respirations (14). Aerobic respiration, generally at the beginning of fermentation, consists of two biochemical reactions: one dominated by spoilage microorganisms and the other by plant cellular respiration (32). These two biochemical reactions utilize the residual air in the material to completely oxidize and breakdown energy substances such as glucose into acetyl coenzyme A through the glycolytic metabolic pathway, catalyzed by enzymes. Then, the dicarbonyl acetyl group of acetyl coenzyme A is converted to CO_2_ and hydrogen atoms through the tricarboxylic acid cycle or the citric acid cycle. Finally, the hydrogen atoms enter the electron transfer chain (respiratory chain) and are passed to oxygen, with which water is produced; at the same time, a large amount of heat is released through the oxidative phosphorylation that accompanies the electron transfer process to produce adenosine triphosphate molecules, which results in the loss of fermented feed (31). The second stage is anaerobic respiration, also known as anaerobic fermentation. Under anaerobic conditions, one molecule of glucose is broken down into two molecules of pyruvate through the glycolytic pathway; a small amount of hydrogen and energy is released during this process. Pyruvic acid can then be converted into lactic acid or broken down into alcohol and CO_2_ under the catalysis of different enzymes (33). During this process, methanogenic bacteria in the soil such as *Methylocaldum gracile*, *Methylococcus capsulatus*, *Methylocystis heyeri*, *Methylocapsa aurea*, and *Methylocella palustris* are also mixed into the material and under strict anaerobic environmental conditions they can utilize the anaerobic oxidation of hydrogen for energy, using CO_2_ as an electron acceptor to produce CH_4_ (34). Therefore, it is important to effectively regulate the anaerobic fermentation to inhibit GHG emissions during aerobic respiration and anaerobic fermentation stages.

The LAB can be classified into homofermentative and heterofermentative LAB according to their fermentation type. Homofermentative LAB convert glucose to lactic acid without CO_2_ production during fermentation (35), but heterofermentative LAB produce large amounts of ethanol and CO_2_ in addition to converting glucose to lactic acid during ensiling, resulting in losses of DM and energy (36). In addition, the nitrate accumulated in forage is mainly reduced to a series of substances such as nitrite and N_2_O by Enterobacteriaceae and *Clostridium perfringens*, which contribute to the emission of GHG (37). In terms of global production scale, the production of fermented stored feeds is huge and increasing year by year. The GHG emissions from fermented feed have far-reaching negative impacts on the efficient use of forage, livestock productivity, and climate change. As shown in Fig. 1, gas production and DM loss rate were the highest in the control, whereas the combined use of LAB and cellulase maximally suppressed GHG emission and reduced DM loss. This indicates that a large amount of GHG will be produced in the natural fermentation process, and feed fermentation can be regulated by using LAB and cellulase, which not only can effectively inhibit the emission of GHG in the anaerobic fermentation process but also is of great significance in reducing the loss of DM and nutrients of fermented feed as well as in promoting the sustainable animal production.

### Microbial diversity and relative abundance of rice straw in anaerobic fermentation

Alpha diversity is a key variable in microbiome studies calculated through a series of rarity analyses and is a measure of microbial diversity in a sample. Alpha diversity depends on the number of different microbial richness (Chao1 and ACE indexes) and the degree to which they are uniformly distributed in terms of diversity (Shannon and Simpson indexes) (38). As shown in Fig. 2, fresh rice straw contains a certain amount of moisture and nutrients, and microorganisms originating from the environment are suitable to grow on its surface, so the alpha diversity of rice straw material is high before fermentation. Previous studies of authors showed that after anaerobic fermentation, the microbial community in the feedstock rapidly evolves from Gram-negative to Gram-positive bacteria. Eventually, only LAB became the dominant community during fermentation and inhibited the colonization of other harmful bacteria, leading to a decrease in the microbial diversity index (7). In this study, all additives used accelerated the microbial turnover process and reduced the alpha diversity compared to the control, with the mixed addition showing the best fermentation effect due to bacterial-enzymatic synergies.

In this study, Venn diagrams were used to visualize the similarity and overlap of microbial community between sample groups of rice straw by analyzing the species and numbers of microorganisms unique or common to different treatments of rice straw. OTUs allow the classification of microbial community and are used to analyze the diversity of common microorganisms in each treatment during fermentation (33). As shown in Fig. 3, the number of OTU was higher in rice straw before ensiling than after ensiling, and the mixed treatment of LAB and cellulolytic enzyme showed the least number of OTU. This reflects the rich diversity of epiphytic microorganisms in fresh rice straw and the changes in microbial diversity after ensiling due to the double selection pressures of acidic and anaerobic environments generated by bacterial-enzyme co-fermentation. This is because acidic and anaerobic conditions not only cause rapid death of aerobic Gram-negative bacteria with thin cell walls but also inhibit the growth of some anaerobic bacteria that are sensitive to acidic environment. These pressures also restrict the colonization of fungi such as yeasts and molds that are not conducive to growth in low pH condition, thus leading to a reduction in microbial diversity while promoting anaerobic fermentation and feed storage.

Changes in microbial community and metabolites produced during ensiling have important implications for the fermentation quality of feeds and the health of livestock (39). As shown in Fig. 4, the main microbial community in the rice straw material used in this study were composed of *E. cloacae* and *P. agglomerans. E. cloacae* is a widely distributed Gram-negative bacteria of the Enterobacteriaceae family, which grows under aerobic or anaerobic conditions and is an opportunistic pathogen associated with both humans and plants (40). These bacteria can cause infections of wounds, respiratory and urinary tract infections, and ESBL-bacteraemia bacteria. *P. agglomerans* is a bacteria belonging to the family Enterobacteriaceae, which is an opportunistic pathogen for patients with infectious diseases and can cause soft tissue or bone and joint infections (41). It is widely distributed on plant surfaces, seeds, fruits, and animal or human feces. So far, these two bacteria have been rarely reported in fermented feed, or total mixed ration should be classified as harmful bacteria in anaerobically fermented feed due to their pathogenicity to both animals and plants. In the future, the dynamics of these two harmful bacteria during enisling and their influence on anaerobic fermentation need to be further investigated.

The relative abundance of *E. cloacae*, uncultured *Clostridium* sp., and *L. plantarum* was higher than the other bacteria in the two control groups of this experiment. *E. cloacae* and *Clostridium* sp. are considered to be harmful bacteria in anaerobic fermentation, both of which can colonize fermentation feed in anaerobic and acidic environments, competing with LAB for nutrients and hindering the growth of LAB (42). In addition, both bacteria can decompose proteins to produce NH_3_-N, thus reducing the fermentation quality and nutritional value of fermented feed (43). The relative abundance of *L. plantarum* in the LAB and AC-alone treatment increased markedly as the dominant bacterial community compared to the control. This is due to the fact that the addition of LAB strains FG1 and TH14, and their cultures FM and TM used in this study increased the initial LAB population in the first stage of anaerobic fermentation, and that these LAB have a strong lactic acid fermentation capacity, producing large amounts of lactic acid in anaerobic conditions and lowering the pH, thus improving the anaerobic fermentation. On the other hand, the addition of cellulolytic enzyme AC can promote the decomposition of plant fiber components into monosaccharides, which provides fermentation substrate for the propagation of LAB and promotes lactic acid fermentation. This is because fresh LAB cultured in grass medium have more activity than inoculant LAB, without the buffer period for LAB growth in freeze-dried preparations, and can grow rapidly after addition in anaerobic condition; thus, the enzyme-bacteria synergistic effect is maximized. The synergistic effect of LAB and AC can effectively promote the glycolysis Embden-Meyerhof pathway (EMP), promote the production of large amounts of lactic acid, and inhibit the growth of harmful microorganisms, thus increasing the abundance of *L. plantarum* and improving the fermentation quality of silage.

### The KEGG pathway in anaerobic fermentation

Anaerobic fermentation is dominated by the activities of microorganisms degrading substrates or transforming metabolites through complex metabolic pathways, and prediction of their metabolic potential assesses the microbial impact on anaerobic fermentation systems (44). The GOM was the most dominant metabolic pathway for microorganisms associated with rice straw material and its fermented feed in this experiment, followed by carbohydrate and amino acid metabolic pathways (Fig. 5). The higher moisture and aerobic environment in the rice straw material favored the growth of aerobic bacteria. After ensiling, enhanced biosynthesis of secondary metabolites and microbial metabolism in a diverse environment due to exuberant proliferation of LAB resulted in a higher proportion of GOM metabolic pathways in fermented feed than in materials and showed that the GM + AC treatment had a stronger GOM metabolic pathway. The LAB enhance carbohydrate metabolic pathways in anaerobic fermentation by inhibiting the proliferation of *Clostridia difficile*, thereby greatly attenuating amino acid metabolic pathways.

### Microbial network and fermentation product

The microorganisms associated with anaerobic fermentation are diverse, and the structure of microbial community and their co-occurrence network systems are important factors affecting the final fermentation product (7). In the early stages of anaerobic fermentation, some air remaining in the feed material creates aerobic condition. At this point, the aerobic bacteria symbiotic with the feedstock become dominant and influence the fermentation quality during the early anaerobic phase. These bacteria consume oxygen and release CO_2_, thus creating an anaerobic environment. Thereafter, the dominant microbial community gradually alternates from Gram-negative to Gram-positive bacteria, with LAB eventually becoming the dominant community. The fermentation of LAB further intensifies the anaerobic and acidic conditions in the feed environment, which not only leads to the death of spoilage microorganisms in the anaerobic environment but also enables long-term preservation of the fermentation feed. As shown in Fig. 6a, the microbial co-occurrence network system is intricate, in which LAB and other microorganisms showed mutual influence and constraint. In this study, a co-occurrence network with *L. plantarum* as the dominant community was formed at 60 days of ensiling of rice straw and showed a positive correlation with *L. curvatus. L. curvatus* is one of the main bacteria distributed in poultry product, dairy product, and fermented meat product, and this bacterium shows good antimicrobial activity against foodborne pathogens, especially *Staphylococcus aureus*, *Staphylococcus epidermidis*, *Escherichia coli*, and *Vibrio vulnificus*, as well as a probiotic effect on animals. *L. curvatus* synergizes with *L. plantarum* during ensiling by converting WSC to lactic acid, which inhibits the growth of harmful bacteria and serves as a regulator of fermentation. In addition, *L. plantarum* and *C. acidophilum* show a mutually restraining relationship. Anaerobic acid-tolerant *C. acidophilum* is usually isolated from acidic peat bog soils. This bacterium is a spore-forming, motile bacillus with flagellum-rich flagella, and its end product is butyric acid produced by degrading glucose (45). These bacteria produce NH_3_-N by degrading proteins and are susceptible to adverse effects on anaerobic fermentation.

*P. agglomerans*, which is negatively correlated with *L. plantarum*, is a Gram-negative parthenogenetic anaerobic bacillus that causes a wide range of opportunistic infections such as septicemia, pneumonia, septic arthritis, wound infections, and meningitis (46). It inhabits a wide range of ecosystems and has the property of breaking down organic compounds and is responsible for the deterioration of food and fermented feed (43). *L. plantarum* rapidly responds to the dual stresses of anaerobic and acidic environments during fermentation by undergoing malolactic fermentation, resulting in a negative correlation that inhibits the growth of *P. agglomerans* (Fig. 6b). In addition, *B. cepacia* and *E. cloacae*, which are positively correlated with pH and NH_3_-N, are both harmful bacteria for anaerobic fermentation. *B. cepacia* is an aerobic non-fermenting, oxidase-positive Gram-negative bacillus (47). It is the causative agent in patients with cystic fibrosis and chronic granulomatous disease and is usually found in natural environments such as soil, water, and plant or in food, where it can survive for several months in moist environment (48). *E. cloacae* is the dominant community in the woody anaerobic fermentation and is frequently distributed in deteriorating and rotting ragweed fermented feed. This bacterium utilizes amino acid decarboxylase to produce biogenic amines such as cadaverine and tyramine and to reduce fermentation quality. In anaerobic fermentation, the vigorous proliferation of *L. plantarum* inhibits the growth of this bacterium, so that it is generally undetectable in high-quality fermented feed (49). *C. pabulibutyricum* and *C. tyrobutyricum* are strictly anaerobic Gram-staining variable *Clostridia*. Both are mostly isolated from high-moisture and low-quality fermented feed and produce butyric acid during fermentation, which is the main strain that reduces feed quality (50). *Clostridia* are usually involved in the degradation of proteins to NH_3_-N, and their main fermentation products are butyric acid, acetic acid, hydrogen, and CO_2_ from various carbohydrates such as glucose and xylose (6), thus forming a positive correlation between the former and NH_3_-N, as well as the latter and acetic acid.

In this study, cleaner anaerobic fermentation of crop straw and GHG emission reduction were thoroughly investigated. The LAB and cellulase microbial additives can exert bacterial-enzyme synergism to form a symbiotic microbial network system dominated by LAB. By modulating microbial GOM and carbohydrate and amino acid metabolic pathways during fermentation, GHG emissions were reduced; feed conversion was increased; and fermented feed quality was improved. This is of great significance for the effective use of crop by-product resources, the promotion of sustainable animal husbandry, and the suppression of global warming.

## MATERIALS AND METHODS

### Fermented feed preparation

Rice (*Oryza sativa* L. cultivar: Hohi) was harvested on 20 September 2022 at Kanagi Experimental Farm of Hirosaki University (Hirosaki, Japan). Rice was harvested at maturity with a harvester (HF441G; Iseki, Matsuyama, Tokyo, Japan), and fresh rice straw was cut into 1- to 2-cm lengths using a chopper (CX-201; Yamamoto Co., Ltd., Tendo, Japan). After thorough mixing, 1-kg samples with three replicates were taken. The samples were placed in ice boxes at 5°C and transported immediately to the laboratory for analysis of chemical composition, energy, macro-mineral, microbial communities, GHG production, and DM loss.

Fermented feed was prepared using 20-L polyethylene experiment silo (Hiryu KN type, Asahi Kasei, Tokyo, Japan), each silo containing approximately 8 kg of rice straw material. Commercial LAB inoculant FG1 (*L. plantarum*) and TH14 (*L. casei*) and cellulase AC (*A. cellulolyticus*) were used in the fermented feed preparation. In order to improve the activity of LAB frozen powder, de Man, Rogosa, and Sharpe (MRS) broth was used for the incubation of FG1 (MF) and TH14 (MT) at 30°C for 24 h. Experiment A was designed as control, FG1, MF, AC, FG1 + AC, and MF + AC, and experiment B was designed as control, TH14, MT, AC, TH14 + AC, and MT + AC. The LAB inoculant was added to the FM at 5 mg/kg according to the recommendation of the bacterial agent producer, with an inoculum count of 1.0 × 10^5^ CFU/g of FM. The inoculated bacteria counts of MF and MT were also adjusted to 1.0 × 10^5^ CFU/g of FM. The AC is mainly composed of dextranase, pectinase, and carboxymethyl cellulase, with a total cellulase activity of 7,350 U/g and a fermented feed addition of 0.01% FM. The LAB inoculant and AC enzyme were dissolved in 2% and 1% distilled water, respectively, and then added by spraying with an electrosprayer (SSP-5H; Fujiwara Sangyo Co. Ltd., Osaka, Japan), and the control was sprayed with an equal amount of sterilized distilled water. Fermented feed was stored at ambient temperature (25°C–36°C), and after 60 days of ensiling, fermented feed samples were removed from the silos and mixed thoroughly before sub-samples were extracted. The sample from each treatment was divided into three parts. The first portion with approximately 50 g of sample was stored in a −80°C freezer for future PacBio single-molecule real-time (SMRT) analysis; the second portion with approximately 200 g of sample was dried for the analysis of chemical composition; and the third portion with approximately 10 g was used for the preparation of a liquid extract to analyze the microbial population and fermented feed fermentation.

### Chemical, energy, and macro-mineral analysis

The material and fermented feed of rice straw were dried in an oven at 65°C for 48 h. The dried samples were ground using a mill (T1-200; CMT Co., Ltd., Tokyo, Japan). The EE, ash, DM, and CP were analyzed using Association of Official Analytical Chemists (AOAC) methods such as 920.39, 923.03, 930.15, and 990.03, respectively (51). The OM content was analyzed based on the weight lost after ashing. Heat-stable amylase and sodium sulfite were used for the NDF and ADF procedure exclusive of residual ash. ADL was measured by solubilization of cellulose with sulfuric acid (52). For the LBC analysis, 10 g of the material was weighed and placed into 90 mL of distilled water and homogenized with Stomacher Lab Blender (400; Seward, UK) for 5 min. After reducing the pH to 4 with 0.1-N HCl, the titration amount required for the pH from 4.0 to 6.0 (mmol/kg DM) with 0.1-N NaOH was used to calculate the LBC (53). The contents of NPN, TP, and NDIP were analyzed by Kjeldahl analysis method (54). The contents of NEm, NEl, and NEg were measured according to the method of the National Research Council (55). The Ca, P, Mg, and K were analyzed by a wet-ashing method and then determined with an atomic absorption spectrophotometer (LAMBDA 1050; PerkinElmer, Connecticut, USA) method (56).

### DM loss and gas production analysis

The emission yield of GHG and DM loss rate were determined using the following method as described by Cai (11). The volume of 100 g of fermented feed material was accurately weighed, packed into plastic bags (information on fermented feed bags), vacuumed, and sealed, then immersed in a water bath at constant temperature (30°C) to measure the increased volume of water. After 60 days of ensiling, the volume of plastic silo was measured again in the same way, and the volume of expanding gases is calculated from the difference with the volume at packing and converted into gas production per kilogram of fresh material. The rate of DM loss during ensiling is calculated from the difference between the weight of DM of the material and that of the fermented feed.

### Microbial population analysis

The plate counting method was used for analysis of microbial population including LAB, aerobic bacteria, coliform bacteria, yeasts, and molds in rice straw before and after ensiling (9). Ten grams of the sample and 90-mL 0.85% sterilized saline solution were homogenized with a Stomacher Lab Blender (400; Seward, UK) for 5 min. The suspension was diluted with saline in a sequence from 10^−1^ to 10^−8^. The 50 μL of diluted suspension was spread on an agar plate. The LAB were incubated and counted on MRS agar (Difco Laboratories, Detroit, MI, USA) in an anaerobic condition by using pack rectangular jar (5 L; Mitsubushi Gas Chemical Company Inc., Tokyo, Japan). The LAB characterization was assigned by the Gram-positive and catalase-negative cocci or rod that mainly formed lactic acid from glucose. Aerobic bacteria and coliform bacteria were cultured on nutrient agar medium (Nissui-Seiyaku Co., Ltd., Tokyo, Japan) and blue light broth agar medium (Nissui-Seiyaku Co., Ltd.) in an aerobic incubator, respectively. White colony counts were for aerobic bacteria, and blue colony counts were for coliform bacteria to distinguish them from aerobic bacteria. All bacterial agar plates were incubated at 30°C for 2–3 days. Yeasts and molds were incubated and counted together on potato dextrose agar medium (Nissui-Seiyaku Co., Ltd.) at 30°C for 3–5 days. Based on cell morphology under the microscope observation, the yeasts and molds were distinguished from other bacteria. The viable microbial counts were shown as microbial colonies in CFU per gram of FM.

### Analysis of fermented feed fermentation

The cold-water extract method was used to analyze the fermentation products of the fermented feed as described by Cai (57). The fermented feed sample (10 g) and sterilized distilled water (90 mL) were homogenized in a beaker and kept in a refrigerator at 4°C for 24 h. Thereafter, the sample extracts were filtered by using quantitative ashless filter paper (circle size: 5A, 110 mm; Advantec Co., Ltd., Tokyo, Japan). The filtrate was used to measure pH, lactic acid, acetic acid, propionic acid, butyric acid, and NH_3_-N. The pH meter (D-71; Horiba Co., Ltd., Kyoto, Japan) was used to determine the pH of the filtrate. The high-performance liquid chromatography (HPLC) system (LC-2000 Plus; JASCO Co., Tokyo, Japan) was used to determine the organic acid (24, 57) of fermented feed filtrate. The HPLC system contained JASCO UV-2070 detector (450 nm), 3-mM HClO_4_ eluent and reagent (0.2-mM bromothymol blue + 8-mM Na_2_HPO_4_ + 2-mM NaOH), and 1-mL/min flow rate, Shodex RSpak column (KC-811; Showa Denko K. K., Tokyo, Japan) in an oven at 60°C. The Kjeltech auto distillation (2200; Foss Tecator, Hoganas, Sweden) was used for the analysis of NH_3_-N as described by Cai (57).

### Bacterial community analysis

#### The DNA extraction

For DNA extraction, 10-g samples of rice straw material and its fermented feed were mixed with 90 mL of 0.85% sterilized saline solution and shaken at 250 rpm with a shaker (FS-003; Tokyo Garasu Kikai, Tokyo, Japan) in a freezer room at 4°C for 45 min. The mixture liquid was filtered through four layers of pre-autoclaved gauze and then centrifuged at 10,000 rpm and 4°C for 10 min to obtain a microbial precipitate from the filtrate. Then the DNA was extracted from the precipitate using a DNA kit (D5625-01; Omega, Norcross, GA, USA) according to the manufacturer’s instruction manual (58). The DNA samples were stored in a deep freezer at −80°C for future analysis.

#### The SMRT sequencing

For SMRT sequencing, the primers 27F and 1492R were used to amplify full-length 16S rRNA gene of DNA sample. The PCR amplicons were purified, quantified, and pooled in equal amounts (58). The purified SMRTbell libraries from the amplified DNA were prepared from the SMRTbell Express Template Prep Kit (version 2.0; PacBio, Menlo Park, CA, USA). The Sequel II Sequencing Kit (version 2.0) was used to sequence the libraries on individual PacBio Sequel II 8M cells.

PacBio RS II instrument (Pacific Biosciences, Menlo Park, CA, USA) was used to perform SMRT sequencing using P6-C4 chemistry. The raw data were processed by the protocol RS_Readsofinsert.1 in PacBio SMRT Portal (version 2.7) software (59). The low-quality sequences were removed by the Quantitative Insights Into Microbial Ecology (QIIME) package (version 1.7). The representative sequences were aligned by the extracted high-quality circular consensus sequencing using 100% clustering of sequence identity. The Python Nearest Alignment Space Termination was used to cluster and classify the inference with U-statistics (UCLUST) analysis. The UCLUST algorithm was used to identity the unique sequences, which classified into OTU based on a 99% threshold. The Chimera Slayer tool was used to remove the potential chimeric sequences in the representative set of OTU. According to classification at an 80% minimum bootstrap threshold, different OTUs and annotations of the taxonomic information for each OTU representative sequence based on Bergey’s taxonomy at the genus, family, order, class, and phylum levels were classified to implement the SILVA database (version 132). If OTUs were present only one or two times, these were removed. QIIME software was used to calculate the alpha diversity including ACE, Chao1, Shannon, and Simpson indexes (60). The different relative abundances of microbial communities at genus and species level were analyzed using Excel Statistical Package for Windows. Following the analysis result of OTU clustering, open-source software package (version 1.2) of R statistical tools was used to produce the Venn diagrams for description of the shared and unique microorganisms in all samples. The functional annotations of sequenced metagenomic sequences through the 16S rRNA marker gene were used to assign the metabolic potential of the microbial community and the composition of functional genes based on the KEGG. The Phylogenetic Investigation of Communities by Reconstruction of Unobserved States (version 2) was used to analyze the functional profiles and differences among different groups. Since the rice straw materials and fermented feed in experiments A and B showed similar microbial community, experiment A was used as a representative for KEGG metabolism pathway analysis. Python language tool was used to draw the relationship figure of microbial network. The circle represents the microorganism species; the circle size represents the average relative abundance of the species; the line represents the correlation between the two species; the thickness of the line represents the strength of the correlation; and the color of the line represents orange as positive correlation and green as negative correlation. The R package pvclust (version 3.0.2) was used to performe the analysis of hierarchical cluster and heat map.

The corresponding value of the middle heat map is the Spearman correlation coefficient *r*, which ranges between −1 and 1; *r* < 0 indicates a negative correlation (blue), and *r* > 0 indicates a positive correlation (red). The symbols “*,” “**,” and “***” represent *P* < 0.05, *P* < 0.01, and *P* < 0.001, respectively.

### Statistical analysis

Analysis of variance (ANOVA) was performed using the general linear model procedure of Statistical Package for the Social Sciences (SPSS version 19.0; SPSS Inc., Chicago, IL, USA) to examine the differences between samples, and significance was declared at *P* < 0.05. The chemical and protein composition, energy, macro-minerals, fermentation quality, DM loss, and gas emission of samples were subjected to one-way ANOVA. Tukey’s honestly significant difference test was employed for different sample means (61).

### Conclusion

Microbial additives can be used to make high-quality feed from rice straw. The LAB and cellulase can exert a synergistic bacterial-enzyme effect, forming a symbiotic microbial network with LAB as the dominant community. By regulating the GOM, carbohydrate, and amino acid metabolic pathways of microorganisms during ensiling, this system not only improves the fermentation quality of feed but also reduces the GHG emission and DM loss. This is important for the effective use of crop residue resources, the promotion of sustainable livestock production, and the suppression of global warming.

### Highlights

- Rice straw silage is an important source of greenhouse gas emission.
- Increased gas production during rice straw silage leads to fermentation loss.
- Microbial additives can modulate microbial co-occurrence network and reduce greenhouse gas emission during silage fermentation.
- Cellulase and lactic acid bacteria exert a synergistic enzyme-bacteria effect for cleaner silage fermentation.

## Data Availability

All sequence reads generated in this study have been deposited to the National Center for Biotechnology Information Sequence Read Archive under BioProject accession number PRJNA1100248.
